# BMPRIA is required for osteogenic differentiation and RANKL expression in adult bone marrow mesenchymal stromal cells

**DOI:** 10.1038/s41598-018-26820-8

**Published:** 2018-05-31

**Authors:** Soma Biswas, Ping Li, Hongguang Wu, Md Shafiquzzaman, Shunichi Murakami, Michael D. Schneider, Yuji Mishina, Baojie Li, Jing Li

**Affiliations:** 1 Bio-X Institutes, Key Laboratory for the Genetics of Developmental and Neuropsychiatric Disorders, Ministry of Education, Shanghai Jiao Tong University, Shanghai, 200240 China; 2 Department of Ophthalmology, XinHua Hospital, Shanghai Jiao Tong University, School of Medicine, Shanghai, China; 3 Department of Orthopaedics, Case Western Reserve University, Cleveland, Ohio, 44106 USA; 4 British Heart Foundation Centre of Research Excellence, National Heart and Lung Institute, Imperial College London, London, W12 0NN UK; 5 Department of Biologic & Materials Sciences, School of Dentistry, University of Michigan, 1011N, University Ave., Ann Arbor, MI 48109 USA

## Abstract

Bone morphogenetic proteins (BMPs) activate the canonical Smad1/5/8 and non-canonical Tak1-MAPK pathways via BMP receptors I and II to regulate skeletal development and bone remodeling. Specific ablation of *Bmpr1a* in immature osteoblasts, osteoblasts, or osteocytes results in an increase in cancellous bone mass, yet opposite results have been reported regarding the underlying mechanisms. Moreover, the role for BMPRIA-mediated signaling in bone marrow mesenchymal stromal cells (BM-MSCs) has not been explored. Here, we specifically ablated *Bmpr1a* in BM-MSCs in adult mice to study the function of BMPR1A in bone remodeling and found that the mutant mice showed an increase in cancellous and cortical bone mass, which was accompanied by a decrease in bone formation rate and a greater decrease in bone resorption. Decreased bone formation was associated with a defect in BM-MSC osteogenic differentiation whereas decreased bone resorption was associated with a decrease in RANKL production and osteoclastogenesis. However, ablation of *Tak1*, a critical non-canonical signaling molecule downstream of BMP receptors, in BM-MSCs at adult stage did not affect bone remodeling. These results suggest that BMP signaling through BMPRIA controls BM-MSC osteogenic differentiation/bone formation and RANKL expression/osteoclastogenesis in adult mice independent of Tak1 signaling.

## Introduction

The bone undergoes life-long remodeling, which is carried out by bone forming osteoblasts and bone resorbing osteoclasts^1–3^. Bone mass and quality can be regulated at the level of osteoblastogenesis/bone formation, osteoclastogenesis/bone resorption, and coupling between bone formation and resorption^4–6^. Several lines of evidence have shown that some osteogenic regulators, e.g., transforming growth factor-β1 (TGF-β1), bone morphogenetic proteins (BMPs), and insulin growth factor-1 (IGF-1), released from bone matrix after resorption or synthesized by osteoclasts are important coupling factors^5,7^. Besides, osteoblasts produce some of the master regulators of osteoclastogenesis, e.g., macrophage colony stimulating factor (M-CSF), receptor activator of nuclear factor-kappaB ligand (RANKL), and osteoprotegerin (OPG)^4,8,9^. Disruption of the coupling between bone formation and resorption can cause bone-related diseases^10^.

BMPs are known as ectopic bone inducers and play crucial roles in skeletal development and bone homeostasis^11–15^. The BMP signaling pathway commences with binding to the heterodimeric serine/threonine kinase receptors, BMPRI and BMPRII^12,16,17^. BMPRII is constitutively active and can phosphorylate and activate BMPRI upon ligand binding. All the BMPRI molecules, BMPRIA, BMPRIB and ACVR1, have been shown to contribute to skeletal development^15,18–21^. It has been reported that *Bmpr1a*, when overexpressed, successfully rescues the differentiation defect of chondrocytes due to deletion of *Bmpr1b*^22^, suggesting that they have redundant functions.

Previous studies have revealed vital roles of BMPs and their receptors in osteoblast and chondrocyte differentiation and bone formation during skeletal development^11,16,18,23^. Conventional knockout mice for *Bmpr1a* show early embryonic lethality^24^. Conditional *Bmpr1a* ablation in osteoclasts and their progenitors uncovered critical roles for BMP and BMPR in osteoclast differentiation and bone resorption as well^25,26^. Interestingly, osteoblast-specific *Bmpr1a* deletion with *osteocalcin-Cre* (*Og2-Cre*) results in age-dependent change in bone mass. The young mutant mice show a low bone mass due to reduced bone formation, while the aged mutant mice show a high bone mass due to reduced bone resorption^21^. Inducible *Bmpr1a* ablation in osteoblasts using *Col1-CreER* mice results in a dramatic decrease in osteoclastic activity through the decrease of RANKL/OPG ratio without affecting osteoblastic activity, leading to an increase in bone mass^21,27–29^. On the other hand, *Bmpr1a* ablation in mature osteocytes does not affect osteoclastogenesis or bone resorption, yet bone formation rate and bone mass are increased^30,31^. Thus, the roles played by BMPR1A at different stages from BM-MSC to osteocyte, especially in BM-MSCs, remains discordant and warrants further investigation. In addition, the physiological roles of Tak1-mediated non-canonical pathways of BMPs in BM-MSCs has not yet been elucidated. Expression of Tak1 is essential for embryonic development and germline deletion of Tak1 leads to embryonic lethality^32^. Earlier studies have reported that Tak1 is critical for normal development of growth plate and joints besides having important regulatory function in innate immunity, hematopoietic stem cell survival, and embryonic angiogenesis as well as in homeostasis of liver, airway smooth muscles, cardiac muscles and cartilages^33–39^. Moreover, Tak1 has been shown to play important roles in osteoclast differentiation and survival, osteoblast maturation, and chondrocyte differentiation^40–44^.

In this study, we generated transgenic *Prx1-CreERT; Bmpr1a*^*f/f*^ and *Prx1-CreERT; Tak1*^*f/f*^ mouse lines to delineate the roles of BMPRIA and Tak1 in adult BM-MSCs, when endochondral ossification becomes minimal, using transgenic *Prx1-CreERT* mice generated in Shunichi Murakami’s lab^45^ that express tamoxifen inducible CreER under the control of a 2.4 kb Prx1 promoter. The *Prx1-CreERT; Bmpr1a*^*f/f*^ mice showed increased bone mass due to decreased bone formation and much greater reduction in osteoclastic resorption. On the other hand, *Prx1-CreERT; Tak1*^*f/f*^ mice showed no significant change in bone mass. Our results suggest that BMPRIA signaling controls BM-MSC osteogenic differentiation and the coupling to osteoclastogenesis in a *Tak1*-independent manner.

## Materials and Methods

### Mice

The *Rosa-tdTomato* mouse line was purchased from the Jackson Laboratory. The transgenic *Prx1-CreERT* mice were generated in Shunichi Murakami’s lab^45^ and the floxed *Bmpr1a* and *Tak1*^*f/f*^ mice were generated in Michael D. Schneider’s lab^33,46^. *Rosa-tdTomato* mice were bred with *Prx1-CreERT* mice to generate *Prx1-CreERT; Rosa-tdTomato* mice. *Bmpr1a*^*f/f*^ and *Tak1*^*f/f*^ mice were mated with *Prx1-CreERT* transgenic mice to generate *Prx1-CreERT; Bmpr1a*^*f/f*^ and *Prx1-CreERT; Tak1*^*f/f*^ mice, in which *Bmpr1a* or *Tak1* was deleted within the osteochondral progenitor cells. Tamoxifen was injected to adult mice to delete the genes. Animals were bred and housed under specific pathogen-free (SPF) condition and provided with food and water *ad libitum*. Animal experimentations in this study were performed following the recommendations of the National Research Council Guide for Care and Use of Laboratory Animals, with all the experimental protocols approved by the Institutional Animal Care and Use Committee of Shanghai Jiao Tong University, China [SYXK (SH) 2011-0112].

### Tamoxifen (TAM) administration

TAM (T5648, Sigma-Aldrich, St. Louis, MO, USA) powder was dissolved in corn oil at a concentration of 10 mg/ml. It was foil-wrapped to protect from light. TAM was administered intraperitoneally to 2-month-old mice at a dose of 100 mg/kg body weight every other day in the early morning for a total of 3 doses. *Rosa-tdTomato* reporter mice were used to evaluate the Cre activity. Femurs, tibiae and lumbar vertebrae were analyzed one month after the last TAM injection by micro-computed tomography (micro-CT), X-ray imaging, and histomorphometry. The same schedule of TAM administration was followed for all the experiments and genotypes.

### X-ray and micro-CT analyses

Radiographs of femurs, lumbar vertebrae from adult mice were taken using the Cabinet X-Ray system (LX-60, Faxitron Bioptics) following a standardized setting (45 kV for 8 s).

After dissecting free of soft tissue, femurs and tibiae from adult (n = 8 for all groups) male mice were fixed overnight in 4% paraformaldehyde (PFA) and stored in 70% ethanol. The distal femurs and proximal tibiae were scanned using a high-resolution Skyscan1176 micro-CT scanner system (Bruker, Kontich, Belgium). Briefly, the following settings for scanning were applied: 50 kV source voltage, 0.2 mm aluminium filter, 270 μA source current, 1350 ms exposure time, 8.96 μm image pixel size and rotation step (degree) of 0.500. After scanning, 3D images were reconstructed using NRecon version 1.6.9.8 (Skyscan, Kontich, Belgium), with a ring artifact reduction of 8, 30% beam hardening correction, and smoothing of 2 using a Gaussian kernel. 2D/3D analyses were performed using CTAn version 1.15.4.0+ (Skyscan, Kontich, Belgium). Finally, CTvox version 2.6.0 r908 (Skyscan, Kontich, Belgium) was used for 3D visualisation. Bone morphometric parameters such as bone volume (BV/TV), trabecular thickness (Tb.Th), trabecular number (Tb.N), trabecular separation (Tb.Sp) were derived following the manufacturer’s instructions. Nomenclature and abbreviations of micro-CT parameters were followed according to guidelines recommended by American Society for Bone and Mineral Research.

### *In vivo* calcein labeling

For fluorochrome-labeled *in vivo* bone studies, mice received calcein (C0875, Sigma-Aldrich, Louis, MO, USA) twice intraperitoneally at a dose of 0.2 mg/20 g body weight at 7-day interval and were sacrificed 1 day after the second injection. The dynamic histomorphometric parameters of bone turnover including mineral apposition rate (MAR), bone formation rate per bone volume (BFR/BV), mineralizing surface per bone surface (MS/BS) were quantified from calcein labeling.

### Bone Histology

For histologic evaluation of the bone, femurs were fixed in 4% PFA overnight, decalcified in EDTA for 3–4 weeks, dehydrated in gradient ethanol, cleared in xylene, and embedded in paraffin. After that, 4 μm thick sections were cut with microtome (RM2255, Leica Microsystems GmbH). For histological analysis, sections were stained with Villanueva-Goldner’s one-step trichrome and Safranin O/Fast green stain.

### Bone histomorphometry

Undecalcified femurs were used as histomorphometric samples. In brief, after anaesthetized mice were euthanized by cervical dislocation, femurs were removed, fixed in 4% PFA overnight, rinsed in 1XPBS for 3 times, dehydrated in an increasing series of alcohol from 95% to 100%, cleared in xylene overnight, vacuumed for 30 min. and then embedded in methyl methacrylate (Merck-Schuchardt). Sections were cut to a thickness of 4 μm using a hard tissue microtome (RM2255, Leica Microsystems GmbH). Dynamic histomorphometry was conducted on unstained bone sections while sections stained with Villanueva-Goldner’s one-step trichrome were used for static histomorphometry. All parameters were measured at the secondary spongiosa of distal femur section where analyses commenced 350 μm distal to the growth plate. Nikon Eclipse 80*i* microscope (Nikon Instruments Inc.) was used for capturing and analyzing images. All bone-specific parameters were measured using OsteoMeasure software (OsteoMetrics Inc.). Units of measurement and expression of these parameters accord with the guidelines of the American Society for Bone and Mineral Research (ASBMR).

### Real-Time Quantitative PCR (qPCR)

Total RNA from cells or femurs were extracted with Trizol reagent (Invitrogen, USA) according to the manufacturer’s instructions. cDNA was synthesized using Transcriptor First Strand cDNA synthesis kit (Roche, Basel, Switzerland) with random anchored-oligo (dT) 18 primers and random hexamer. qPCR analyses were performed using FS Universal SYBR Green Master Premix (Roche). Reactions were run in triplicate in three repeated experiments in a total volume of 20 μl with Light Cycler 480 Instrument II (Roche). The relative expression levels of mRNA species were quantified using the delta-delta Ct (∆∆Ct) method. GAPDH (glyceraldehyde 3-phosphate dehydrogenase) was used as endogenous control gene to normalize the expression of all target genes. The primer sequences used for real-time PCR are listed below:

*Opg* F: 5′-CACCCTGTGTGAAGAGGCCT-3′

R: 5′-GCAGGCTCTCCATCAAGGCA-3′

*M-Csf* F: 5′-CTGACACAGGCCATGTGGAG-3′

R: 5′-GAGAGGGTAGTGGTGGATGT-3′

*Rankl* F: 5′-GCA CAC CTC ACC ATC AAT GCT-3

R: 5′-GGT ACC AAG AGG ACA GAG TGA CTT TA-3′

*Alp* F: 5′-TGAGCGACACGGACAAGA-3′

R: 5′-GGCCTGGTAGTTGTTGTGAG-3′

*Osx* F: 5′-ACTCATCCCTATGGCTCGTG-3′

R: 5′-GGTAGGGAGCTGGGTTAAGG-3′

*Ocn* F: 5′AGCAGGAGGGCAATAAGGTAGT-3′

R: 5′-ACCGTAGATGCGTTTGTAGGC-3′

*Runx2* F: 5′-TTTAGGGCGCATTCCTCATC-3′

R: 5′-TGTCCTTGTGGATTAAAAGGACTTG-3′

*Trap* F: 5′-TGTCATCTGTGAAAAGGTGGTC-3′

R: 5′-ACTGGAGCAGCGGTGTTATG-3′

*Bmpr1a* F: 5′-TCATGTTCAAGGGCAGAATCTAGA-3′

R: 5′-GGCAAGGTATCCTCTGGTGCTA-3′

*Gapdh*F: 5′-CCACAGTCCATGCCATCAC-3′

R: 5′-CATACCAGGAAATGAGCTTGAC-3′

### Western blot analysis

Cells or bone tissues were homogenized in T-PER reagent (Thermo Fisher Scientific) supplemented with protease and phosphatase inhibitors. Protein concentration was determined using a BCA Protein Assay Kit (Thermo Fisher Scientific). 20 μg of protein lysates of each sample was subjected to SDS-PAGE (8–12%) and transferred onto a nitrocellulose membrane. Immunoblots were incubated with the specific primary antibodies overnight at 4 °C with gentle shaking. After being washed three times for 15 min each with TBST (Tris-buffered saline, 0.1% Tween 20), blots were incubated with horseradish peroxidase (HRP)-conjugated secondary antibody (Sigma) for one hour. Next, the protein level was detected with Super Signal West Femto Maximum Sensitivity Substrate (Thermo Fisher Scientific) and visualized by FluorChem E system (Protein Simple, CA, USA). The following antibodies were used: p-Smad1/5/8 (Cell Signaling, 9511), Tak1 (Cell Signaling, 4505, Boston, MA), Bmpr1a (Abcam, ab38560), β-Actin (Santa Cruz, Sc-47778).

### Measurement of urine Deoxypyridinoline (DPD)

Urinary DPD level was determined to evaluate the bone resorption rate *in vivo*, following the procedure recommended by MicroVue (Quidel, CA, USA). For this study, mice were individually housed and urine samples were collected early in the morning using a microcapillary tube. DPD values were normalized to the urine levels of creatinine.

### TRAP (tartrate-resistant acid phosphatase) staining

Paraffin sections (4 μm) were stained using the Acid Phosphatase, Leukocyte TRAP Kit (387 A, Sigma-Aldrich) following the manufacturer’s instructions. Images were taken using the Nikon Eclipse 80*i* microscope (Nikon Instruments Inc.). Cells with ≥3 nuclei were considered TRAP-positive multinuclear osteoclasts.

### Isolation and culture of bone marrow BM-MSCs

Briefly, after sacrificing the adult mice, whole body was rinsed in 70% (v/v) ethanol for 2–3 min. Femurs and tibiae were dissected and carefully cleaned from muscle, connective tissue and residual soft tissues. Bones were then transferred to a sterile culture dish containing α-MEM supplemented with 1× penicillin/streptomycin on ice. Two ends of each bone were excised and a syringe needle (27-gauge) was inserted into bone cavity to flush out bone marrow with α-MEM. This process was repeated several times until the color of the bones turned from red to white. To get rid of any bone debris, the cell suspension was filtered through a 70 μm nylon mesh filter (BD Falcon, USA). Cells were cultured in cell culture plate in α-MEM supplemented with 10% fetal bovine serum (FBS; Sigma), 100 μg/ml penicillin and 100 μg/ml streptomycin (Gibco). Plates were then incubated at 37 °C with 5% CO_2_ in a humidified chamber for 5 days. Non-adherent cells were removed by changing the medium and the BM-MSCs were either directly used or kept frozen for future experiments.

### CFU (Colony forming unit) assay

To enumerate the number of MSCs in bone marrow, the CFU assay was performed. Cells were seeded in 12-well plates at the density of 5 × 10^6^ per well. The plates were incubated in a humidified 5% CO_2_/37 °C environment. Fresh complete α-MEM medium was changed every 3 days. After 10 days of culture, the formation of CFUs was evaluated by washing the plates with sterile phosphate buffered saline (PBS) twice, fixing with 4% PFA for 30 min at room temperature, and staining with ALP (Sigma). Colonies with more than 50 cells were counted using light microscopy.

### Osteogenic differentiation of BM-MSCs

For alkaline phosphatase (ALP) activity assay, BM-MSCs were plated at a density of 5 × 10^4^ cells/well in 12-well plates containing complete medium. After 24 hr., culture medium was replaced with osteogenic differentiation medium containing complete medium supplemented with 10 mM β-glycerol phosphate and 50 μg/ml Ascorbic acid for 7–10 days. Medium was changed every 3 days. To evaluate osteogenic differentiation, cells were then washed with sterile 1X PBS, fixed in 4% PFA for 30 min at room temperature, and stained for ALP using an Alkaline Phosphatase Kit (sigma) following the manufacturer’s protocol.

### Statistical analysis

Results were expressed as means ± SD. Comparisons between wild-type and transgenic mice were analyzed using two-tailed unpaired Student’s t-test and a *p* value of less than 0.05 was considered to indicate statistical significance. 3–4 litters were analyzed for every parameter. Each experiment was repeated 3–4 times using materials from different mice to ensure the reproducibility of the experimental findings. For *in vitro* experiments, 3–4 pairs of mice were used. For bone histomorphometry and micro-CT analyses, 8 mutants and 8 control littermates were used.

### Data Availability

Data supporting the findings of this study are available from the corresponding author on request (email: libj@sjtu.edu.cn).

## Results

### Tracing of Prx1 lineage cells in the adult bone

**Figure 1:**
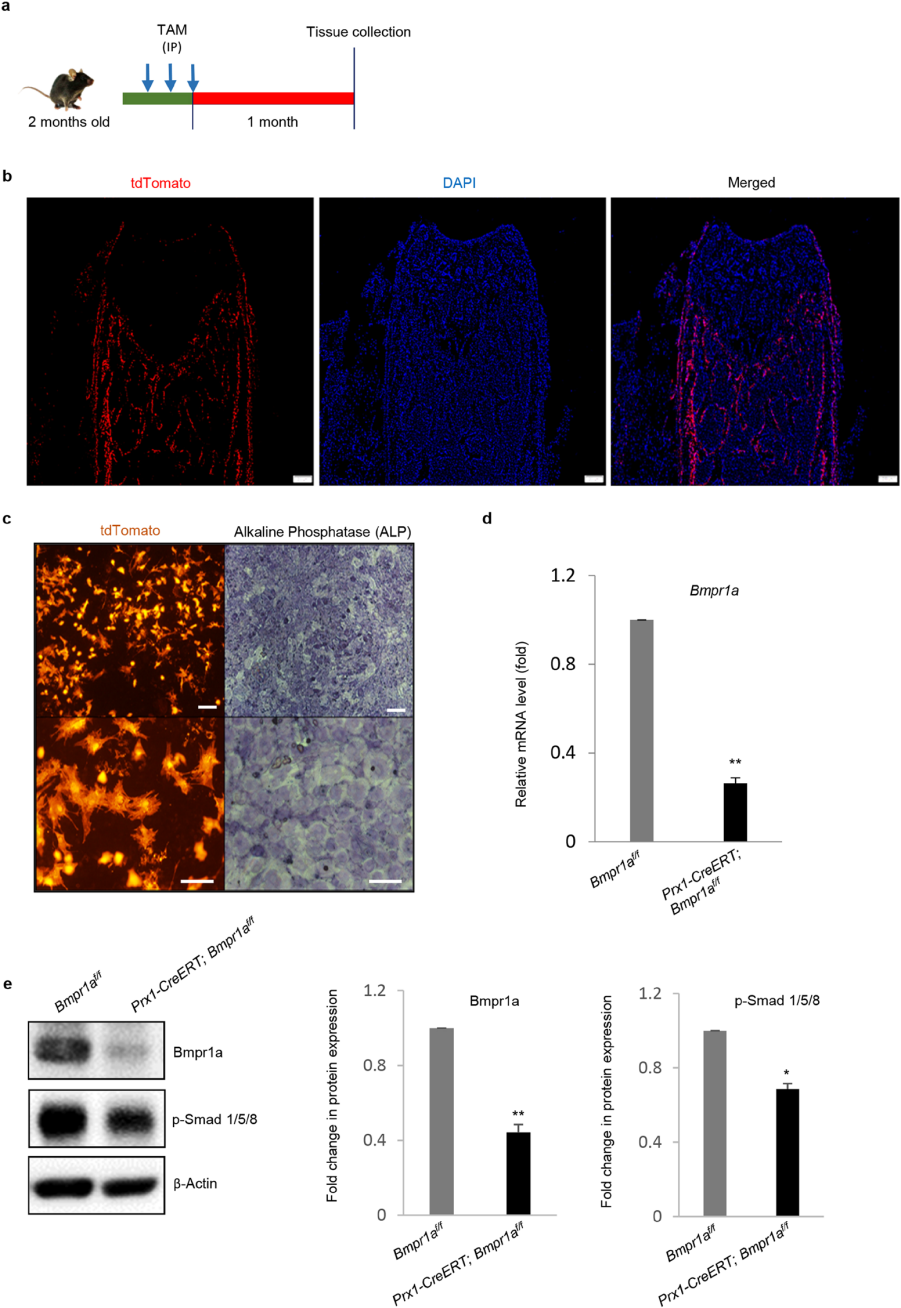
*Prx1-CreERT* mediated tracing and Bmpr1a ablation in adult mice. (**a**) Schedule for TAM administration to 2-month-old mice. TAM was injected to both *Prx1-CreERT; Bmpr1a*^*f/f*^ and *Bmpr1a*^*f/f*^ mice (age- and sex-matched littermates) on every other day for 3 doses. One month after the last Tam injection, bone tissues were retrieved and analyzed. (**b**) Tissue specificity and effectiveness of Cre recombinase in *Prx1-CreERT; Rosa-tdTomato* mice. Osteoblasts and osteocytes as well as the endosteal and periosteal surfaces of bones were labeled by tdTomato fluorescent signals. N = 3 mice, Scale bar = 200 μm. (**c**) *In vitro* analysis revealed tdTomato + bone marrow BM-MSCs (left panel) with the potential to differentiate into ALP-positive osteoblasts (right panel). Lower panel indicates higher magnification of upper panel. Upper panel scale bar = 100 μm and lower panel scale bar = 50 μm. (**d**) Quantitative real-time RT-PCR analysis of BM-MSCs showed that *Bmpr1a* was deleted (>70%) in the mutant mice. (**e**) Immunoblotting of bone extracts also confirmed a reduction in the level of Bmpr1a and phosphorylated-Smad 1/5/8. Left panel: Representative Western blots; Right panel: Quantification of relative protein expressions (from three repeated experiments) plotted in the graph as fold change. N = 4 mice/group; ^*^P < 0.05. For the full-length blots see Supplementary Fig. S1.

In an attempt to trace Prx1 lineages in adult bone, we generated *Prx1-CreERT; Rosa-tdTomato* mice. We administrated TAM to adult mice through intraperitoneal route. Similar to previously reported results^45^, we detected tdTomato fluorescence signals in osteoblasts and osteocytes in cancellous and cortical bone, as well as cells at the endosteal and periosteal surfaces (Fig. 1a,b), yet, the absence of tdTomato signals in growth plate or articular cartilage suggests that Prx1 + BM-MSCs do not generate chondrocytes in adult mice (Fig. 1b). In addition, we isolated bone marrow BM-MSCs from *Prx1-CreERT; Rosa-tdTomato* mice (injected with 3 doses of TAM) and cultured them *in vitro*. A large portion of the cells were Tomato positive and they could differentiate into ALP positive osteoblasts (Fig. 1c). These results confirmed that Prx1 + BM-MSCs are present in the bone marrow and can give rise to osteoblasts and osteocytes, but not chondrocytes, in adult mice.

### *Bmpr1a* ablation resulted in increased bone mass

To evaluate the functions of BMPR1A in adult BM-MSCs, we generated *Prx1-CreERT; Bmpr1a*^*f/f*^ mice. TAM was administrated to 2-month-old *Prx1-CreERT; Bmpr1a*^*f/f*^ mice 3 times and the mouse bones were analyzed one month after the last TAM injection. Quantitative RT-PCR analysis of bone extracts revealed a significant reduction in the levels of *Bmpr1a* mRNA in the mutant mice (Fig. 1d). Western blot analysis of bone extracts also confirmed a decrease in Bmpr1a and phosphorylated Smad 1/5/8 in the mutant mice (Fig. 1e and Supplementary Fig. S1). These results demonstrated that the TAM administration deleted *Bmpr1a* in the adult bone.

During the studies, we found that even 3 doses of TAM showed an effect on bone mass in adult wild type mice. TAM appeared to increase bone mass at the cancellous bone (Supplementary Fig. S2a and b). To exclude the possible complication of TAM, we used *Bmpr1a*^*f/f*^ treated with TAM as a control for *Prx1-CreERT; Bmpr1a*^*f/f*^ mice treated with TAM. *Prx1-CreERT; Bmpr1a*^*f/f*^ and *Bmpr1a*^*f/f*^ mice without TAM treatment were also included as controls. X-Ray analysis of femurs from *Prx1-CreERT; Bmpr1a*^*f/f*^ and WT littermates showed no difference in the radiodensity without TAM injection (Supplementary Fig. S3a). Similar findings were found when we examined femurs histologically (Supplementary Fig. S3b). Therefore, *Prx1-CreERT; Bmpr1a*^*f/f*^ mice have no baseline phenotype, validating the effectiveness of tamoxifen-inducible CreERT system.

**Figure 2:**
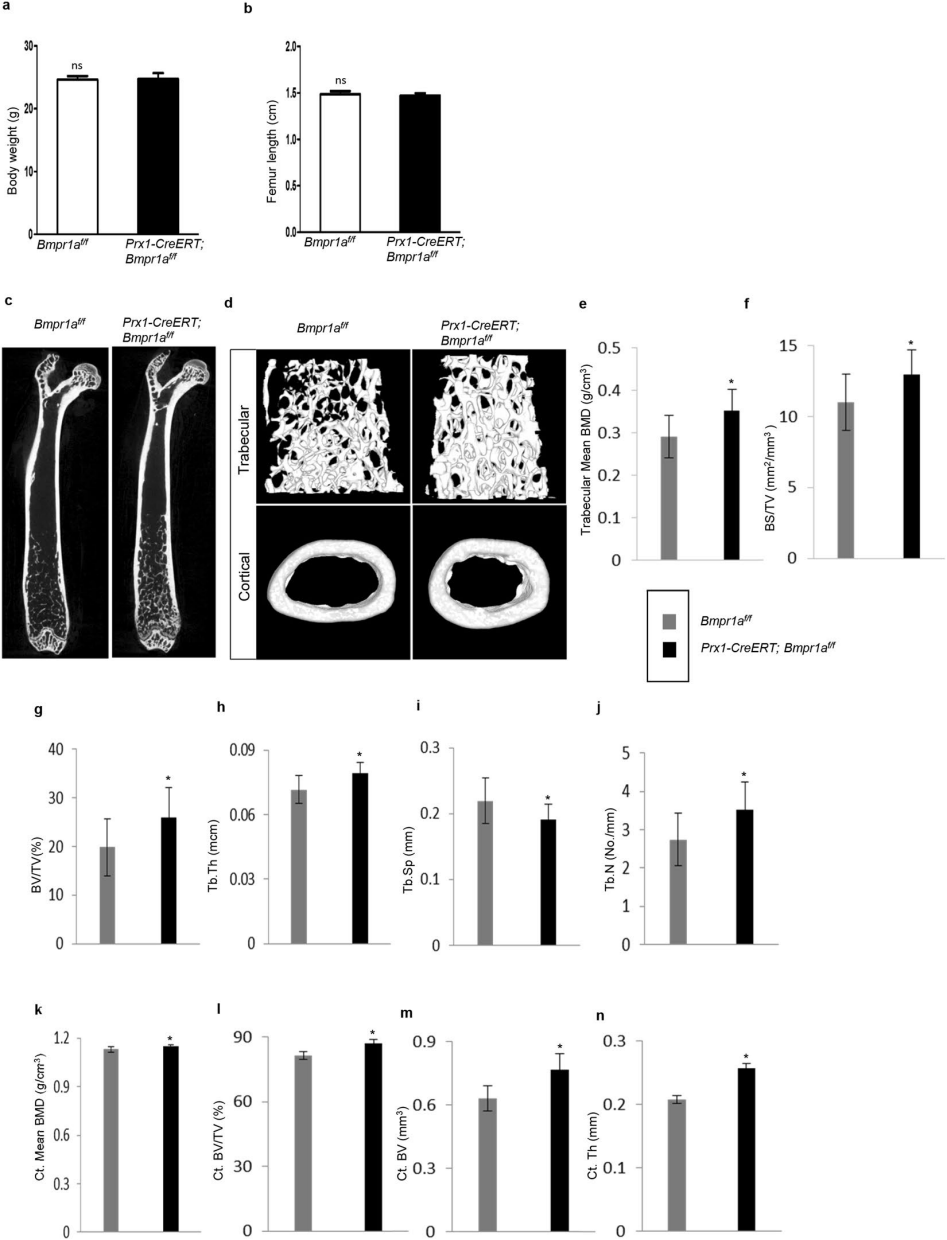
Micro-CT-derived bone morphometry results revealed an increase in bone volume and bone mineral density in *Prx1-CreERT; Bmpr1a*^*f/f*^ mice. (**a**,**b**) Body weight and femur lengths of mutant and control littermates showed no significant difference. Data are expressed as mean ± SEM from 8 mice per group. (**c**) Representative micro-CT images of 2D whole femur segital section from adult *Prx1-CreERT; Bmpr1a*^*f/f*^ and their respective control mice. (**d**) 3D distal femoral metaphyseal cancellous bone (upper panel) and mid-diaphyseal cortical bone (lower panel). N = 8 mice per group. (**e**–**j**) Micro-CT quantification of bone histomorphometry indices revealed high trabecular bone mass in mutant mice. Tb. BMD, trabecular bone mineral density (g/cm^3^) (**e**); BS/TV, bone surface/tissue volume (mm^2^/mm^3^) (**f**); BV/TV, bone volume/tissue volume (%) (**g**); Tb.Th, trabecular thickness (mcm) (**h**); Tb.Sp, trabecular spacing (mm) (**i**); and Tb.N, trabecular number (No./mm) (**j**). (**k**–**n**) Micro-CT quantification revealed high cortical bone phenotypes in mutant mice. Ct. BMD, Cortical bone mineral density (g/cm^3^) (**k**); Ct, BV/TV, cortical bone mass (%) (**l**); Ct. BV, cortical bone volume (mm^3^) (**m**); and Ct.Th, cortical thickness (mm) (**n**). N = 8 mice per genotype; ^*^P < 0.05.

We found that one month after *Bmpr1a* was deleted in Prx1 + BM-MSCs, the mutant mice looked normal morphologically with no significant difference in body weight or femur length compared with control (Fig. 2a,b). Quantitative data obtained from micro-CT of femurs showed an increase in bone volume and bone mineral density in *Prx1-CreERT; Bmpr1a*^*f/f*^ mice at the cancellous bone (Fig. 2c–j). Unlike other reported osteoblast-specific *Bmpr1a* knockout mouse lines, *Prx1-CreERT; Bmpr1a*^*f/f*^ mice showed an increase in bone volume and bone mineral density at the cortical bone (Fig. 2k–n). Moreover, qualitative analysis of femurs by von-Kossa staining also revealed an increase in the amount of mineralized bone in mutant mice (Supplementary Fig. S4a). On the other hand, the lumbar vertebrae in the mutant mice did not show a significant change compared to control mice (Supplementary Fig. S4b), indicating that Prx1 is not expressed in vertebral bones.

**Figure 3:**
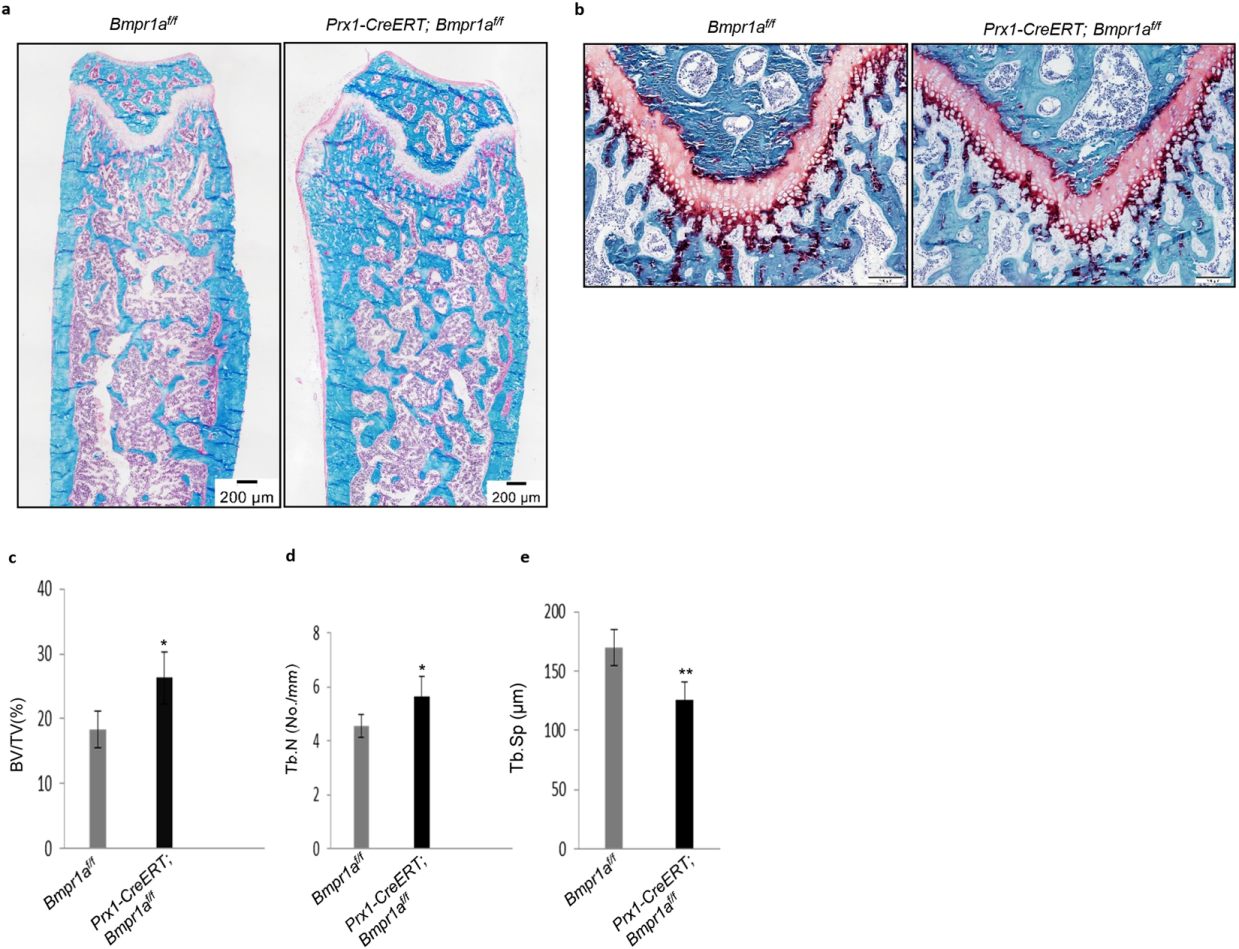
Static histomorphometric analysis showed deletion of *Bmpr1a* resulted high bone mass in adult *Prx1-CreERT; Bmpr1a*^*f/f*^ mice. (**a**) Villanueva-Goldner’s one-step trichrome staining on undecalcified femurs demonstrated the micro-architecture of bone. Scale bar = 200 μm. (**b**) Safranin O staining showed no significant differences in the growth plates of mutants and their respective controls. Scale bar = 100 μm. (**c**–**e**) Quantification using osteomeasure software. BV/TV, bone volume/tissue volume (%) (**c**); and Tb/N, trabecular number (No./mm) (**d**) were increased but Tb.Sp (μm) (**e**) was decreased in *Prx1-CreERT; Bmpr1a*^*f/f*^ mice compared with *Bmpr1a*^*f/f*^ control littermates. Data are mean ± SEM (N = 8 bones/group; 4 fields/bone); ^*^P < 0.05; ^**^P < 0.01.

To validate these findings, we carried out bone histomorphometric analysis of Villanueva-Goldner’s one-step trichrome-stained undecalcified femurs. The long bones of the mutant mice showed normal growth plates (Fig. 3a,b), yet, the bone volume (BV/TV, %) and trabeculae number (Tb.N, mm) were markedly increased whereas trabecular spacing (Tb.Sp, mm) was decreased in the mutant compared to control mice (Fig. 3c–e), similar to the micro-CT results. These results collectively indicate that ablation of *Bmpr1a* in Prx1 + BM-MSCs led to an increase in bone mass.

### Disruption of *Bmpr1a* led to a decrease in bone formation and resorption

**Figure 4:**
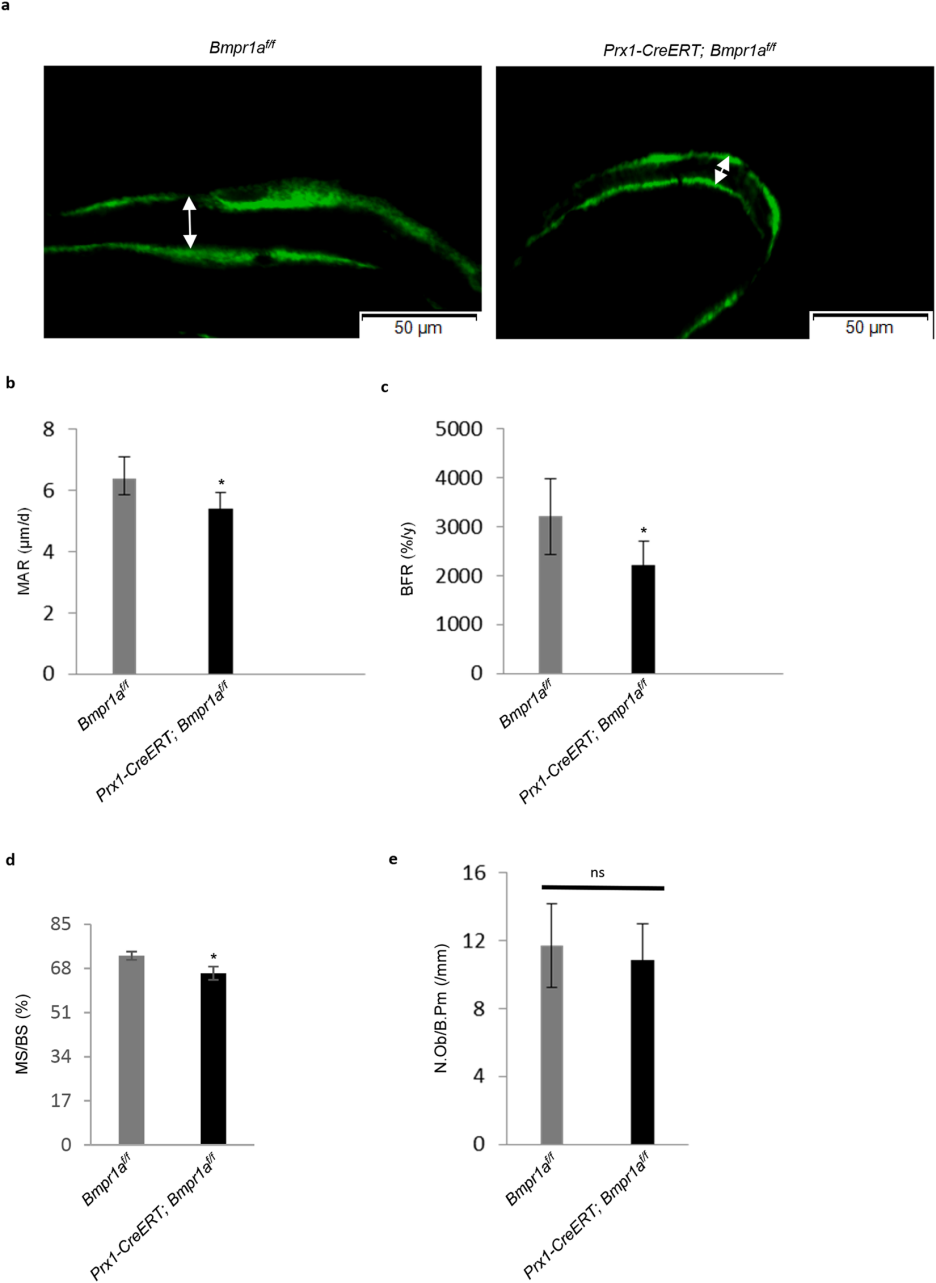
Dynamic morphometric markers for bone formation parameters in adult *Prx1-CreERT; Bmpr1a*^*f/f*^ mice. (**a**) Representative calcein-labeled images of the distal femurs. The distance between two layers of calcein labels visualized by fluorescence represented bone formation that occurred during the 5-d period between calcein injections. Scale bar = 50 μm. (**b**–**d**) Adult *Prx1-CreERT; Bmpr1a*^*f/f*^ mice showed a significant decrease in MAR, mineral apposition rate (μm/d) (**b**), BFR, bone formation rate (%/y) (**c**) and MS/BS, mineralizing surface per bone surface (%) (**d**) values compared with age-matched control mice. Data are mean ± SEM (N = 8 bones/group; 5 fields/bone); ^*^P < 0.05. (**e**) *Prx1-CreERT; Bmpr1a*^*f/f*^ mice revealed no change in the number of osteoblasts when measured the value of one histomorphometric index of bone formation, N.Ob/B.Pm, osteoblast number per bone perimeter (/mm). N = 8 mice. ns = Non-significant.

A harmonized balance between bone formation and resorption is crucial to maintain bone mass. We then analyzed bone formation and resorption rates in *Bmpr1a* deficient bones. Bone formation parameters, MAR, BFR, and MS/BS of the cancellous bone of femurs were assessed by dynamic histomorphometry (Fig. 4a). We found a significant reduction in MAR, BFR, and MS/BS parameters in bone sections of the mutant mice compared to control mice, indicating an overall decrease in bone formation rate (Fig. 4b–d). However, no significant change in the numbers of osteoblasts at the cancellous bone was observed in the mutant mice compared to control mice (Fig. 4e), suggesting that the decrease in the bone formation rate is likely caused by reduced activities of the mutant osteoblasts.

**Figure 5:**
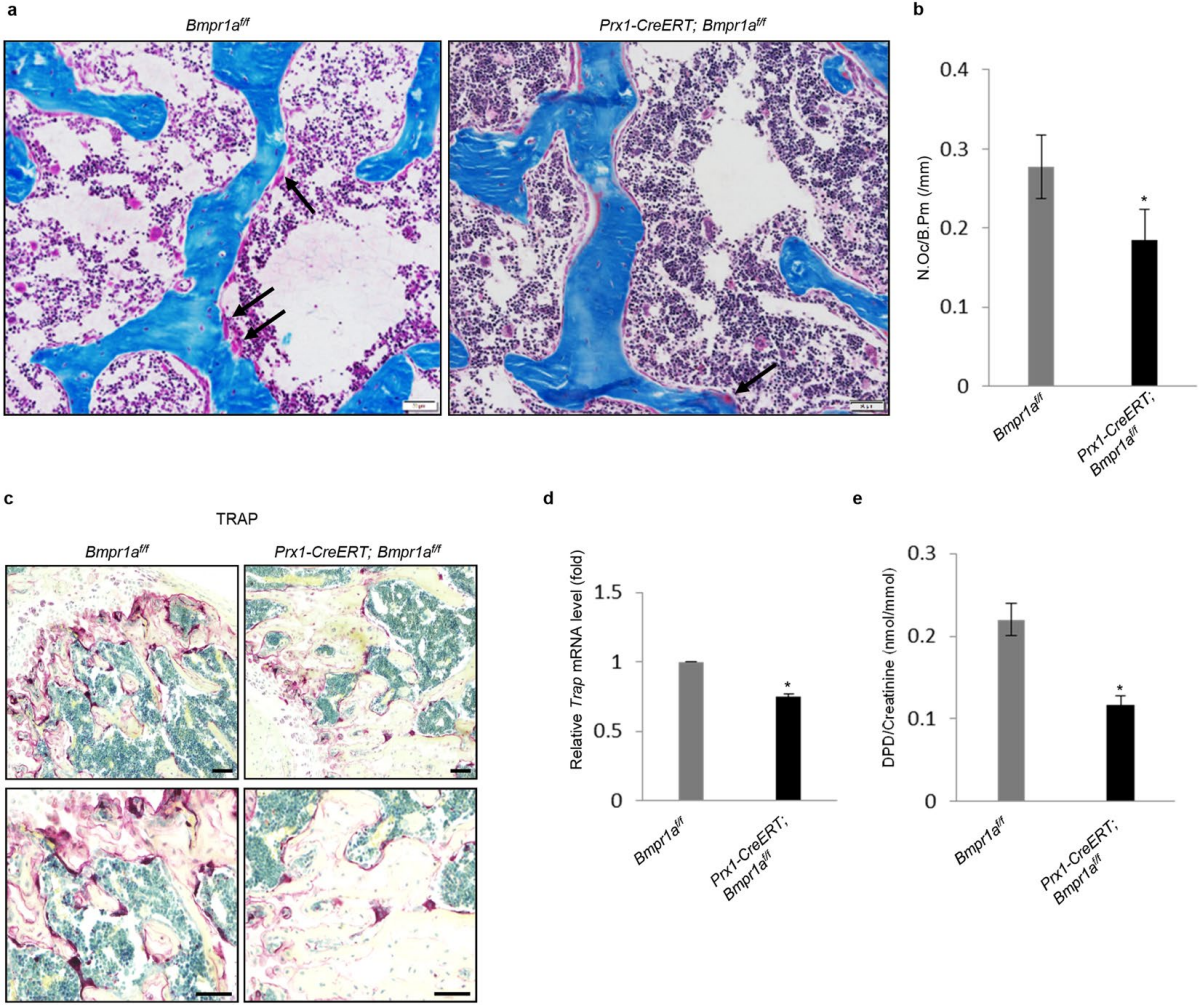
*Bmpr1a* ablation resulted in significant reduction in the bone resorption parameters in adult *Prx1-CreERT; Bmpr1a*^*f/f*^ mice. (**a**) Histological sections of the femurs of adult mice where arrows identified osteoclasts lining cancellous bone surfaces. N = 8 bones/group, Scale Bar = 50 μm. (**b**) Measurements of N.Oc/B.Pm (Number of osteoclast/bone perimeter, /mm) on Villanueva-Goldner’s Trichrome-stained femurs. *Prx1-CreERT; Bmpr1a*^*f/f*^ mice showed a reduction in the number of osteoclasts. ^*^P < 0.05. (**c**) Representative images of TRAP-stained bones of mutant and control littermates exhibited a decrease in the TRAP staining in *Prx1-CreERT; Bmpr1a*^*f/f*^ mice compared to control mice. Scale bar = left panel 100 μm and right panel 50 μm. (**d**) Quantitative RT-PCR analysis of *Trap* as bone resorption marker. mRNA was isolated from bone extract and realtime quantitative RT-PCR was carried out to determine the level of *Trap*. N = 4 mice; ^*^P < 0.05. (**e**) *Prx1-CreERT; Bmpr1a*^*f/f*^ mice showed a decrease in DPD (deoxypyridinoline) level. The ELISA assay was performed using urine samples from mutant and control mice. N = 4 mice per group; ^*^P < 0.05.

In addition, the number of osteoclasts per bone surface calculated from static histomorphometric analysis on Villanueva-Goldner’s one-step trichrome-stained femurs was reduced in the mutant mice compared to control mice (Fig. 5a,b). To validate this finding, we stained the femur sections for tartrate-resistant acid phosphatase (TRAP), an osteoclast marker. The mutant mice showed a decrease in TRAP staining (Fig. 5c), yet we found that it was hard to count TRAP-positive cells on these bone sections. We then measured the mRNA levels of *Trap* of the bone homogenates and found that the mutant mice showed a decrease in the levels of *Trap* (Fig. 5d). Furthermore, we examined the levels of urinary deoxypyridinoline (DPD), an *in vivo* bone resorption marker and found that the mutant mice showed decreased DPD levels (Fig. 5e). Taken together, these results suggest that both bone formation and resorption in adult mice were declined by the deficiency of *Bmpr1a* gene in BM-MSCs, yet the decrease in bone resorption was greater than the decrease in bone formation rate.

### *Bmpr1a*^−/−^ BM-MSCs showed impaired osteoblast differentiation

**Figure 6:**
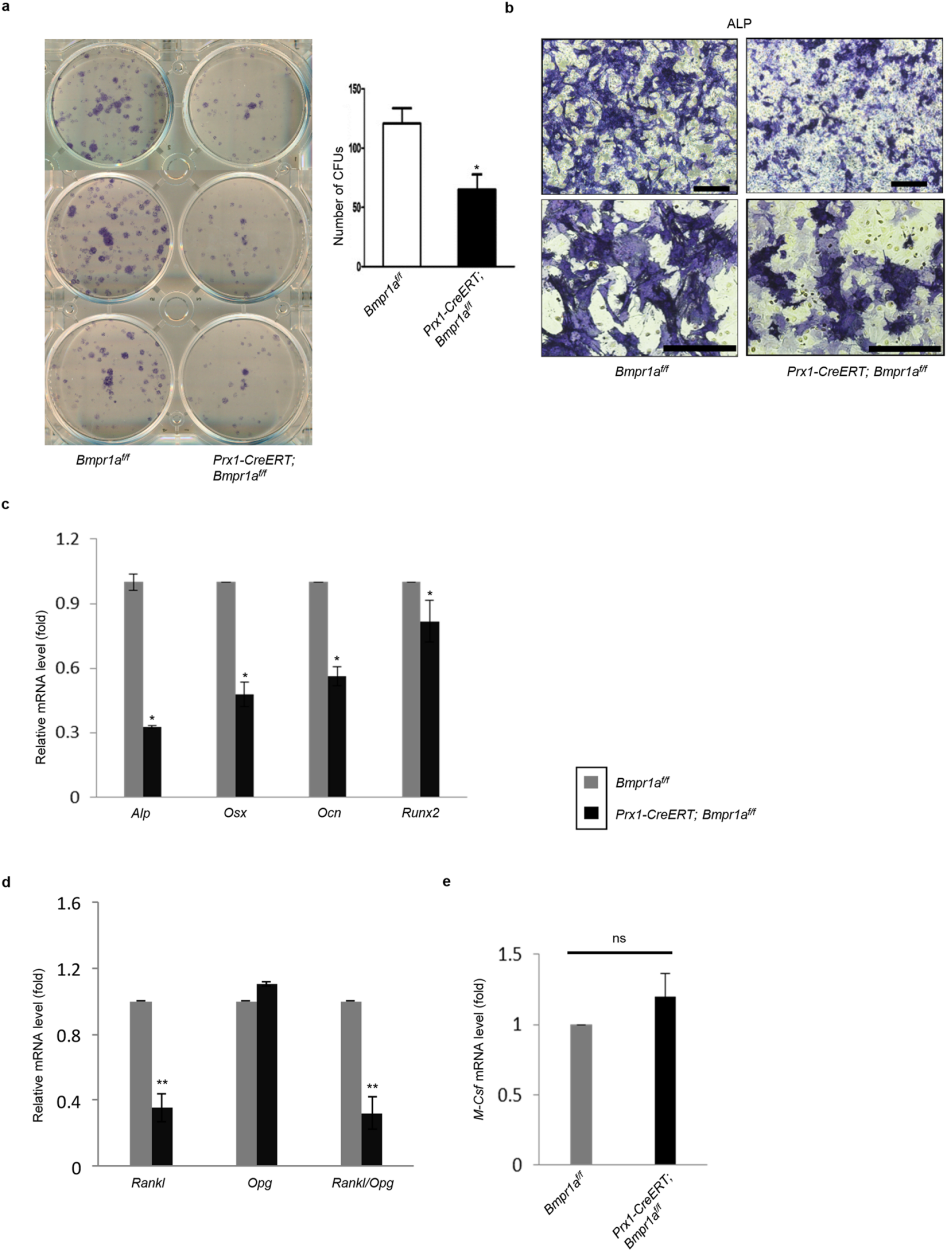
Deletion of *Bmpr1a* in BM-MSCs resulted in impaired osteoblast differentiation and decreased RANKL expression. (**a**) Comparison of bone marrow cell cultures isolated from *Bmpr1a* deficient mice and their respective littermates to evaluate ALP-positive CFU. *Prx1-CreERT; Bmpr1a*^*f/f*^ mice showed a decrease in the number of CFUs. Left panel: ALP staining. Right panel: quantitation data. N = 3 mice; ^*^P < 0.05. (**b**) Alkaline phosphatase (ALP) activity in osteogenic medium showed a decrease in osteoblast differentiation in *Bmpr1a* ablated mice. N = 3. Scale bar = Upper panel 200 μm, lower panel 100 μm. (**c**) Quantitative RT-PCR analysis showed that the expression levels of some osteoblast marker genes, alkaline phosphatase (*Alp*), osterix (*Osx*), osteocalcin (*Ocn*) and runt-related transcription factor 2 (*Runx2*) were decreased in mutant mice. N = 3 mice. (**d-e**) Quantitative RT-PCR analysis of *Rankl*, *Opg* and *M-Csf* mRNA. Results showed that the levels of *Rankl* was down-regulated, while the expression of *Opg* was not affected, resulting in a significant reduction of the ratio of *Rankl* to *Opg* in *Prx1-CreERT; Bmpr1a*^*f/f*^ mice at adult stage. N = 4 mice; ^*^P < 0.05, ^**^P < 0.01 (d). *Bmpr1a* deleted BM-MSCs showed no significant alteration in the expression level of *M-Csf*. N = 4 mice. ns = Non-significant (e).

To determine whether low bone formation rate in the mutant mice was related to osteoblasts defects, we examined the role of *Bmpr1a* in BM-MSC osteogenic differentiation. We harvested bone marrow BM-MSCs to carry out *in vitro* CFU assay and found that deletion of *Bmpr1a* led to significantly reduced number of ALP-positive CFUs, consistent with histomorphometric results in *Prx1-CreERT; Bmpr1a*^*f/f*^ mice (Fig. 6a). We also cultured BM-MSCs in osteoblast differentiation medium and found that inactivation of the *Bmpr1a* impaired osteoblast differentiation, manifested by a decrease in ALP staining and the mRNA levels of *Alp*, *Osx*, *Runx2*, and *Ocn* (Fig. 6b,c). These results, together with the findings that Col1-CreERT-mediated *Bmpr1a* ablation did not affect osteoblast differentiation whereas *Bmpr1a* ablation in mature osteocytes increased bone formation^29,31^, suggest that BMPR1A is required for MSC osteogenic differentiation at an early stage, which is likely through regulating the expression of *Runx2* and *Osx*^23^.

Furthermore, we checked mRNA levels of *Rankl*, *Opg*, and *M-Csf* in BM-MSC cultures by quantitative RT-PCR. These factors are synthesized by BM-MSCs, osteoblasts, and osteocytes to regulate osteoclastogenesis. It was found that the levels of *Rankl* was down-regulated (Fig. 6d), while the levels of *Opg* and *M-Csf* were not significantly affected by BMPRIA deficiency (Fig. 6d,e). This resulted in a significant decrease in the ratio of *Rankl* to *Opg*, consistent with the decrease in the number of osteoclasts and in bone resorption observed in *Prx1-CreERT; Bmpr1a*^*f/f*^ mice.

### Ablation of *Tak1* in Prx1 + BM-MSCs showed no significant bone phenotype

**Figure 7:**
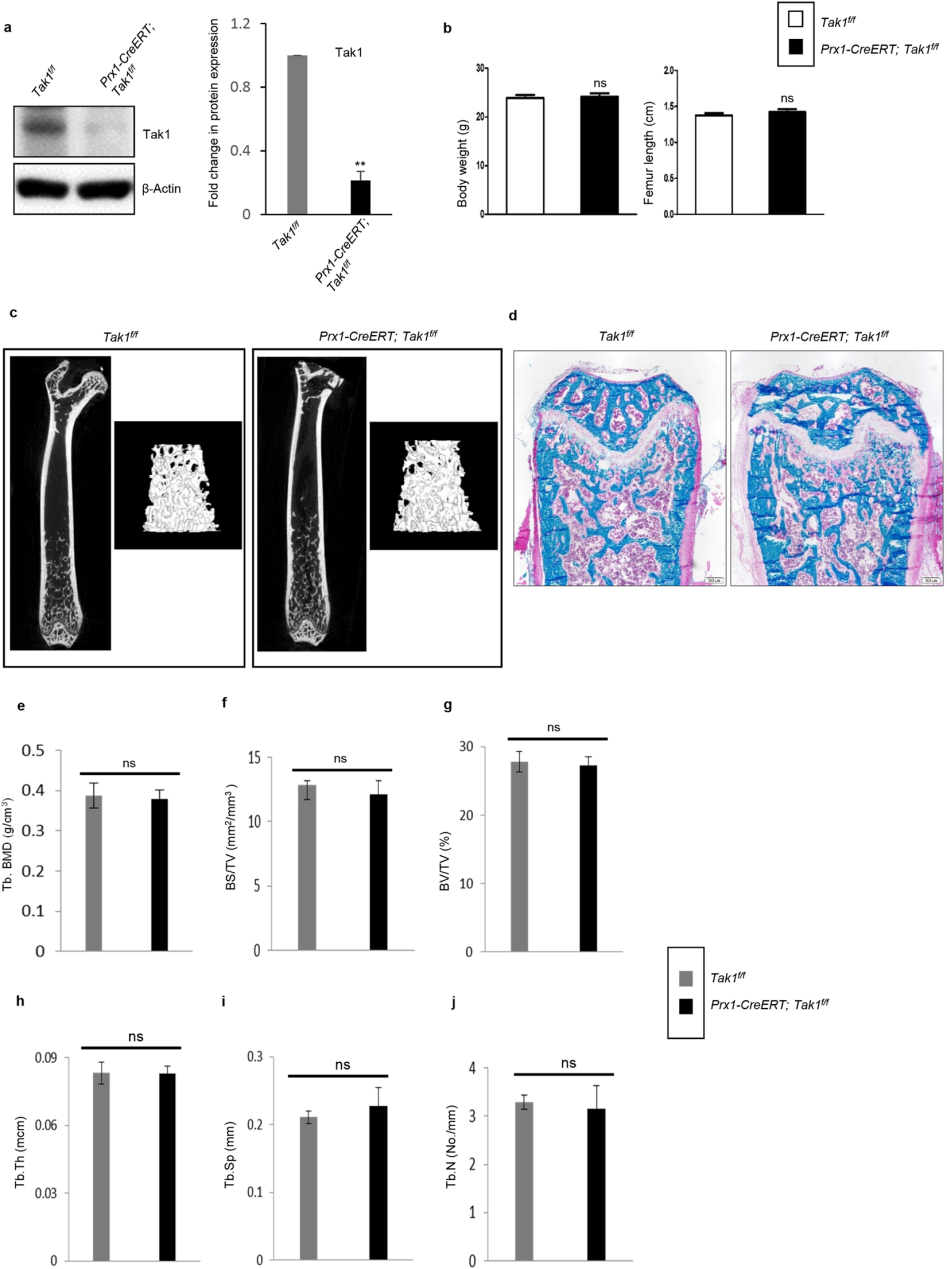
*Tak1* deletion in adult BM-MSCs showed no significant bone phenotype. (**a**) Western blot analysis of bone extracts confirmed a significant reduction in the level of Tak1 in *Prx1CreERT; Tak1*^*f/f*^ mice. Left panel: Representative Western blots; Right panel: Quantification of relative protein expressions (from three repeated experiments) plotted in the graph as fold change. N = 4 mice/group; ^*^P < 0.05; ^**^P < 0.01. For the full-length blots see Supplementary Fig. S5. (**b**) Graph showing no significant difference in body weight, and femur length of mutant and control (age- and sex-matched) mice at adult age. ns = Non-significant. N = 8 per group. (**c**) Micro-CT scanning was performed to analyze cancellous and cortical bone architecture and mineralization on femurs of mice. No significant change between *Prx1CreERT; Tak1*^*f/f*^ *and Tak1*^*f/f*^ was observed. N = 6 mice per group. (**d**) Villanueva-Goldner’s one-step trichrome staining on undecalcified femurs showed the micro-architecture of bone. Scale bar = 200 μm. N = 6 mice per group. (**e**–**j**) Micro-CT results of the femurs. Tb. BMD, trabecular bone mineral density (g/cm^3^) (**e**); BS/TV, bone surface/tissue volume (mm^2^/mm^3^) (**f**); BV/TV, bone volume/tissue volume (%) (**g**); Tb.Th, trabecular thickness (mcm) (**h**); Tb.Sp, trabecular spacing (mm) (**i**); and Tb.N, trabecular number (No./mm) (**j**). ns = Non-significant.

To determine the roles of the non-canonical pathways of BMPs in adult BM-MSCs, we chose to ablate *Tak1*, which encodes a kinase that acts upstream the MAPKs and NF-κB pathways in response to BMPs and TGFβ^47–49^. We generated *Prx1-CreERT; Tak1*^*f/f*^ mice and induced *Tak1* deletion in adult mice by injection of 3 doses of TAM (Fig. 7a and Supplementary Fig. S5). Body weight and femur length in these mice were unchanged (Fig. 7b). The mice were sacrificed one month after the last TAM injection. Micro-CT and X-ray analysis of long bones were performed and no significant change in bone mass at the cancellous and cortical bone was observed between mutant mice and WT littermates (Fig. 7c and e–j and Supplementary Fig. S6a–d). The growth plate of *Prx1-CreERT; Tak1*^*f/f*^ mice was not altered either (Fig. 7d).

To validate the *in vivo* findings, we carried out *in vitro* experiments using BM-MSC from *Prx1-CreERT; Tak1*^*f/f*^ and *Tak1*^*f/f*^ mice. We isolated BM-MSCs to perform CFU assay and found no significant difference in the number of ALP-positive CFUs between mutant mice and control littermates, consistent with micro-CT and X-ray results (Supplementary Fig. S7a). Next, we checked mRNA levels of *Rankl* and *Opg* in BM-MSC cultures by quantitative RT-PCR and found that the levels of *Rankl* and *Opg* and the ratio of *Rankl* to *Opg* were not significantly affected by TAK1 deficiency (Supplementary Fig. S7b and data not shown). These results indicate that *Tak1* in BM-MSCs may not play an essential role in adult mice.

## Discussion

In this study, we generated BM-MSC-specific *Bmpr1a* knockout mice in an attempt to gain an understanding of the effects of BMPRIA-mediated signaling on bone remodeling in adult mice. Note that *Prx1-CreERT*-mediated *Bmpr1a* ablation only occurs in BM-MSCs and their descendent cells in adult mice, when endochondral ossification is completed. This is in contrast to *Dmp1-Cre* and *Osteocalcin-Cre* mediated *Bmpr1a* ablation, which occurs in early embryonic development, and *Col1-CreERT* mediated *Bmpr1a* ablation, which occurs in differentiated osteoblasts^45^. Our results show that although the numbers of osteoblasts are not altered in *Prx1-CreERT; Bmpr1a*^*f/f*^ mice, their function is compromised. This could be attributable to the differentiation defect and the status of maturity of osteoblasts. We cannot exclude the possibility that BMP-BMPR signaling plays a role in the activity of osteoblasts. Our genetic results also show that BMPRIA plays a role in BM-MSC production of RANKL and coupling to osteoclastogenesis. Moreover, the cortical bone phenotypes observed in *Prx1-CreERT; Bmpr1a*^*f/f*^ mice suggest that Prx1 may be a better marker to label periosteal and endosteal BM-MSCs or osteoblasts.

Comparison of our BM-MSC-specific *Bmpr1a* and previously reported osteoblast/osteocyte-specific *Bmpr1a* knockout mouse lines reveals the following similarities and differences. First, adult deletion of *Bmpr1a* in Prx1 + BM-MSCs (*Prx1-CreERT; Bmpr1a*^*f/f*^ mice) results in an increase in bone mass, this is similar to aged *Osteocalcin-Cre; Bmpr1a*^*f/f*^ mice, adult *Col1-CreERT; Bmpr1a*^*f/f*^ mice, and *Dmp1-Cre; Bmpr1a*^*f/f*^ mice^21,29,31^. *Prx1-CreERT; Bmpr1a*^*f/f*^ mice also showed an increase in cortical bone mass, whereas *Dmp1-Cre; Bmpr1a*^*f/f*^ mice showed a reduction in cortical bone. Secondly, *Prx1-CreERT; Bmpr1a*^*f/f*^ mice showed an increase in bone resorption, this is similar to aged *Osteocalcin-Cre; Bmpr1a*^*f/f*^ mice and *Col1-CreERT; Bmpr1a*^*f/f*^ mice, but different from *Dmp1-Cre; Bmpr1a*^*f/f*^ mice, which showed no alterations in osteoclastogenesis and bone resorption. Thirdly, *Prx1-CreERT; Bmpr1a*^*f/f*^ mice showed a decrease in bone formation, this is similar to *Osteocalcin-Cre; Bmpr1a*^*f/f*^ mice and *Col1CreERT; Bmpr1a*^*f/f*^ mice, but opposite to *Dmp1-Cre; Bmpr1a*^*f/f*^ mice (Supplementary Table 1). In general, ablation of *Bmpr1a* in BM-MSCs or osteoprogenitor/osteoblasts gives rise to similar phenotypes in bone mass, bone formation, and bone resorption, which are different from ablation of *Bmpr1a* in terminally differentiated osteocytes.

The discrepancy among these mouse models can be explained in several ways. First, while Cre-mediated *Bmpr1a* ablation affects bone development in embryos, postnatal bone growth, and bone remodeling in adult mice, TAM-induced *Bmpr1a* ablation in adult Prx1 + BM-MSCs affects only bone remodeling. Moreover, due to the closure of the growth plates, TAM-induced *Bmpr1a* ablation in adult Prx1 + BM-MSCs does not go through endochondral ossification, instead, it reflects a direct BM-MSC to osteoblast differentiation. The increase in cortical bone mass, which does not require endochondral ossification, also support that BMPRIA plays a positive role in BM-MSC to osteoblast differentiation. Secondly, production of osteocytes from BM-MSCs is a multi-step process. Cells at various stages are different in their proliferation and differentiation potentials, and their contact with osteoclasts. Thus, their behaviors in response to BMPs may be stage-specific. Indeed, our previous studies have shown that Tsc1 and p38 MAPK have stage-specific effects on osteoblastogenesis and coupling to osteoclastogenesis^50,51^. Lastly, a recent study showed that *Dmp1* could label cell types other than osteocytes including neurons and gut stromal cells^52^. It cannot be excluded that BMPRIA signaling in these cells may have an effect on bone remodeling.

Previous studies have demonstrated the importance of the canonical BMP-Smad1/5/8 pathway in osteoblastogenesis and bone formation. Ablation of *Smad1* in osteoblasts using *Col1-Cre* led to osteopenia^53^. Tak1 is an important signaling molecule of the non-canonical pathway of BMPs. Downstream of Tak1, there are NF-κB and MAPKs pathways, which have been shown to affect osteoblastogenesis^40^. Previous studies have shown that *Tak1* is required for chondrocyte differentiation and survival and for osteoblast differentiation^54–56^. However, TAM-induced *Tak1* ablation in adult Prx1 + BM-MSCs appears not to affect bone mass. One possible explanation is that *Tak1* deficiency may simultaneously impair both NF-κB signaling and MAPK signaling, which have been shown to have opposite functions in regulating osteoblast differentiation^57–61^. Nevertheless, these results suggest that the coordination or even integration of other signals to the BMP-signaling pathways may be crucial in regulating the activity of BM-MSCs, as previously reported^62^.

BMPs and BMPRs are targets for treatment of bone-related diseases^15,63^. Our genetic studies based on BM-MSC-specific *Bmpr1a* and *Tak1* knockout models revealed critical roles of BMPRIA but not Tak1 in adult BM-MSC biology and reinforced the coupling between BM-MSCs and osteoclastogenesis.

## Electronic supplementary material

Supplementary Information

## References

[1] Rachner TD, Khosla S, Hofbauer LC (2011). Osteoporosis: now and the future. Lancet..

[2] Zaidi M, Buettner C, Sun L, Iqbal J (2012). Minireview: The link between fat and bone: does mass beget mass?. Endocrinology..

[3] Baldridge D, Shchelochkov O, Kelley B, Lee B (2010). Signaling pathways in human skeletal dysplasias. Annu Rev Genomics Hum Genet..

[4] Karsenty G (2008). Transcriptional control of skeletogenesis. Annu Rev Genomics Hum Genet..

[5] Crane JL, Cao X (2014). Bone marrow mesenchymal stem cells and TGF-beta signaling in bone remodeling. J Clin Invest..

[6] Engin F, Lee B (2010). NOTCHing the bone: insights into multi-functionality. Bone..

[7] Pederson L, Ruan M, Westendorf JJ, Khosla S, Oursler MJ (2008). Regulation of bone formation by osteoclasts involves Wnt/BMP signaling and the chemokine sphingosine-1-phosphate. Proc Natl Acad Sci USA.

[8] Edwards JR, Mundy GR (2011). Advances in osteoclast biology: old findings and new insights from mouse models. Nat Rev Rheumatol..

[9] Kearns AE, Khosla S, Kostenuik PJ (2008). Receptor activator of nuclear factor kappaB ligand and osteoprotegerin regulation of bone remodeling in health and disease. Endocr Rev..

[10] Michael H, Harkonen PL, Vaananen HK, Hentunen TA (2005). Estrogen and testosterone use different cellular pathways to inhibit osteoclastogenesis and bone resorption. J Bone Miner Res..

[11] Chen G, Deng C, Li YP (2012). TGF-beta and BMP signaling in osteoblast differentiation and bone formation. Int J Biol Sci..

[12] Grafe, I., *et al*. TGF-beta Family Signaling in Mesenchymal Differentiation. *Cold Spring Harb Perspect Biol*. (2017). 10.1101/cshperspect.a022202 PMC5932590 28507020

[13] Yang W (2013). Bmp2 in osteoblasts of periosteum and trabecular bone links bone formation to vascularization and mesenchymal stem cells. J Cell Sci..

[14] Shu B (2011). BMP2, but not BMP4, is crucial for chondrocyte proliferation and maturation during endochondral bone development. J Cell Sci..

[15] Yu PB (2008). BMP type I receptor inhibition reduces heterotopic [corrected] ossification. Nat Med..

[16] Sakaki-Yumoto M, Katsuno Y, Derynck R (2013). TGF-beta family signaling in stem cells. Biochim Biophys Acta..

[17] Wang W, Rigueur D, Lyons KM (2014). TGFbeta signaling in cartilage development and maintenance. Birth Defects Res C Embryo Today..

[18] Pan H (2017). BmpR1A is a major type 1 BMP receptor for BMP-Smad signaling during skull development. Dev Biol..

[19] Shi C (2016). Deletion of BMP receptor type IB decreased bone mass in association with compromised osteoblastic differentiation of bone marrow mesenchymal progenitors. Sci Rep..

[20] Lowery JW (2015). Loss of BMPR2 leads to high bone mass due to increased osteoblast activity. J Cell Sci..

[21] Mishina Y (2004). Bone morphogenetic protein type IA receptor signaling regulates postnatal osteoblast function and bone remodeling. J Biol Chem..

[22] Kobayashi T, Lyons KM, McMahon AP, Kronenberg HM (2005). BMP signaling stimulates cellular differentiation at multiple steps during cartilage development. Proc Natl Acad Sci USA.

[23] Wu M, Chen G, Li YP (2016). TGF-beta and BMP signaling in osteoblast, skeletal development, and bone formation, homeostasis and disease. Bone Res..

[24] Mishina Y, Suzuki A, Ueno N, Behringer RR (1995). Bmpr encodes a type I bone morphogenetic protein receptor that is essential for gastrulation during mouse embryogenesis. Genes Dev..

[25] Okamoto M (2011). Conditional deletion of Bmpr1a in differentiated osteoclasts increases osteoblastic bone formation, increasing volume of remodeling bone in mice. J Bone Miner Res..

[26] Li A (2017). Pharmacologic Calcitriol Inhibits Osteoclast Lineage Commitment via the BMP-Smad1 and IkappaB-NF-kappaB Pathways. J Bone Miner Res..

[27] Kamiya N (2010). Wnt inhibitors Dkk1 and Sost are downstream targets of BMP signaling through the type IA receptor (BMPRIA) in osteoblasts. J Bone Miner Res..

[28] Kamiya N (2008). Disruption of BMP signaling in osteoblasts through type IA receptor (BMPRIA) increases bone mass. J Bone Miner Res..

[29] Kamiya N (2008). BMP signaling negatively regulates bone mass through sclerostin by inhibiting the canonical Wnt pathway. Development..

[30] He G (2017). Differential involvement of Wnt signaling in Bmp regulation of cancellous versus periosteal bone growth. Bone Res..

[31] Lim J (2016). Dual function of Bmpr1a signaling in restricting preosteoblast proliferation and stimulating osteoblast activity in mouse. Development..

[32] Jadrich JL, O’Connor MB, Coucouvanis E (2006). The TGF beta activated kinase TAK1 regulates vascular development *in vivo*. Development..

[33] Xie M (2006). A pivotal role for endogenous TGF-beta-activated kinase-1 in the LKB1/AMP-activated protein kinase energy-sensor pathway. Proc Natl Acad Sci USA.

[34] Ajibade AA, Wang HY, Wang RF (2013). Cell type-specific function of TAK1 in innate immune signaling. Trends Immunol..

[35] Inokuchi S (2010). Disruption of TAK1 in hepatocytes causes hepatic injury, inflammation, fibrosis, and carcinogenesis. Proc Natl Acad Sci USA.

[36] Morioka S (2012). TAK1 kinase signaling regulates embryonic angiogenesis by modulating endothelial cell survival and migration. Blood..

[37] Pera T, Sami R, Zaagsma J, Meurs H (2011). TAK1 plays a major role in growth factor-induced phenotypic modulation of airway smooth muscle. Am J Physiol Lung Cell Mol Physiol..

[38] Tang M (2008). TAK1 is required for the survival of hematopoietic cells and hepatocytes in mice. J Exp Med..

[39] Zhang D (2000). TAK1 is activated in the myocardium after pressure overload and is sufficient to provoke heart failure in transgenic mice. Nat Med..

[40] Yu B (2014). Wnt4 signaling prevents skeletal aging and inflammation by inhibiting nuclear factor-kappaB. Nat Med..

[41] Swarnkar G, Karuppaiah K, Mbalaviele G, Chen TH, Abu-Amer Y (2015). Osteopetrosis in TAK1-deficient mice owing to defective NF-kappaB and NOTCH signaling. Proc Natl Acad Sci USA.

[42] Qi B (2014). Ablation of Tak1 in osteoclast progenitor leads to defects in skeletal growth and bone remodeling in mice. Sci Rep..

[43] Lamothe B, Lai Y, Xie M, Schneider MD, Darnay BG (2013). TAK1 is essential for osteoclast differentiation and is an important modulator of cell death by apoptosis and necroptosis. Mol Cell Biol..

[44] Gao L (2013). TAK1 regulates SOX9 expression in chondrocytes and is essential for postnatal development of the growth plate and articular cartilages. J Cell Sci..

[45] Kawanami A, Matsushita T, Chan YY, Murakami S (2009). Mice expressing GFP and CreER in osteochondro progenitor cells in the periosteum. Biochem Biophys Res Commun..

[46] Mishina Y, Hanks MC, Miura S, Tallquist MD, Behringer RR (2002). Generation of Bmpr/Alk3 conditional knockout mice. Genesis..

[47] Adhikari A, Xu M, Chen ZJ (2007). Ubiquitin-mediated activation of TAK1 and IKK. Oncogene..

[48] Shim JH (2005). TAK1, but not TAB1 or TAB2, plays an essential role in multiple signaling pathways *in vivo*. Genes Dev..

[49] Yamaguchi K (1995). Identification of a member of the MAPKKK family as a potential mediator of TGF-beta signal transduction. Science..

[50] Cong Q (2016). p38alpha MAPK Regulates Lineage Commitment and OPG Synthesis of Bone Marrow Stromal Cells to Prevent Bone Loss under Physiological and Pathological Conditions. Stem Cell Reports..

[51] Wu H (2017). Bone Size and Quality Regulation: Concerted Actions of mTOR in Mesenchymal Stromal Cells and Osteoclasts. Stem Cell Reports..

[52] Lim J, Burclaff J, He G, Mills JC, Long F (2017). Unintended targeting of Dmp1-Cre reveals a critical role for Bmpr1a signaling in the gastrointestinal mesenchyme of adult mice. Bone Res..

[53] Wang M (2011). Smad1 plays an essential role in bone development and postnatal bone formation. Osteoarthritis Cartilage..

[54] Shim JH (2009). TAK1 is an essential regulator of BMP signalling in cartilage. EMBO J..

[55] Gunnell LM (2010). TAK1 regulates cartilage and joint development via the MAPK and BMP signaling pathways. J Bone Miner Res..

[56] Yumoto K (2013). TGF-beta-activated kinase 1 (Tak1) mediates agonist-induced Smad activation and linker region phosphorylation in embryonic craniofacial neural crest-derived cells. J Biol Chem..

[57] Chang J (2009). Inhibition of osteoblastic bone formation by nuclear factor-kappaB. Nat Med..

[58] Greenblatt MB, Shim JH, Glimcher LH (2013). Mitogen-activated protein kinase pathways in osteoblasts. Annu Rev Cell Dev Biol..

[59] Novack DV (2011). Role of NF-kappaB in the skeleton. Cell Res..

[60] Rodriguez-Carballo E, Gamez B, Ventura F (2016). p38 MAPK Signaling in Osteoblast Differentiation. Front Cell Dev Biol..

[61] Yao Z (2014). NF-kappaB RelB negatively regulates osteoblast differentiation and bone formation. J Bone Miner Res..

[62] Kua HY (2012). c-Abl promotes osteoblast expansion by differentially regulating canonical and non-canonical BMP pathways and p16INK4a expression. Nat Cell Biol..

[63] Baud’huin M (2012). A soluble bone morphogenetic protein type IA receptor increases bone mass and bone strength. Proc Natl Acad Sci USA.

