# Supplementary material for: BMPRIA is required for osteogenic differentiation and RANKL expression in adult bone marrow mesenchymal stromal cells
Source: Sci Rep. 2018 May 31;8:8475. doi: 10.1038/s41598-018-26820-8 (PMC5981611; doi:10.1038/s41598-018-26820-8)
Supplement: Supplementary file 1 — Supplementary Information [file 41598_2018_26820_MOESM1_ESM.pdf]

Supplementary Information

BMPRIA is required for osteogenic differentiation and RANKL expression in adult bone marrow mesenchymal stromal cells

Soma Biswas[1,2], Ping Li[1], Hongguang Wu[1], Md Shafiquzzaman[1], Shunichi Murakami[3], Michael D. Schneider[4], Yuji Mishina[5], Baojie Li[1*], and Jing Li[2*]

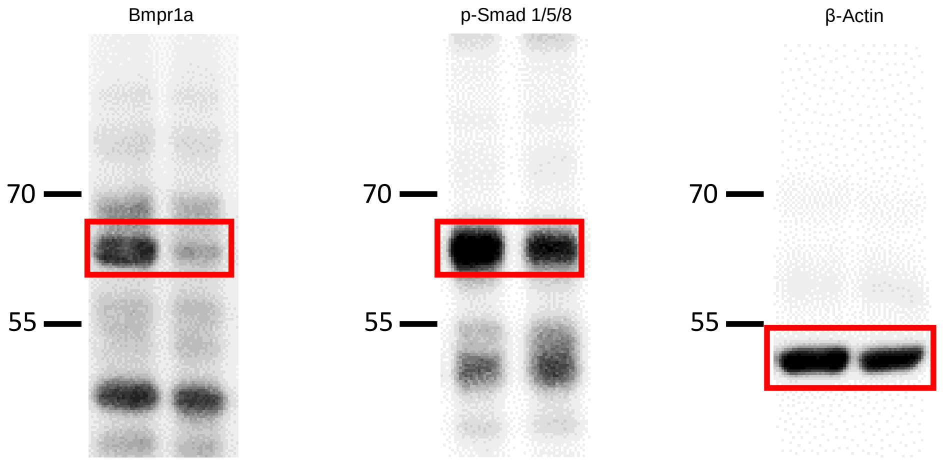

Supplementary Fig. S1: Full-length blot for Fig. 1e

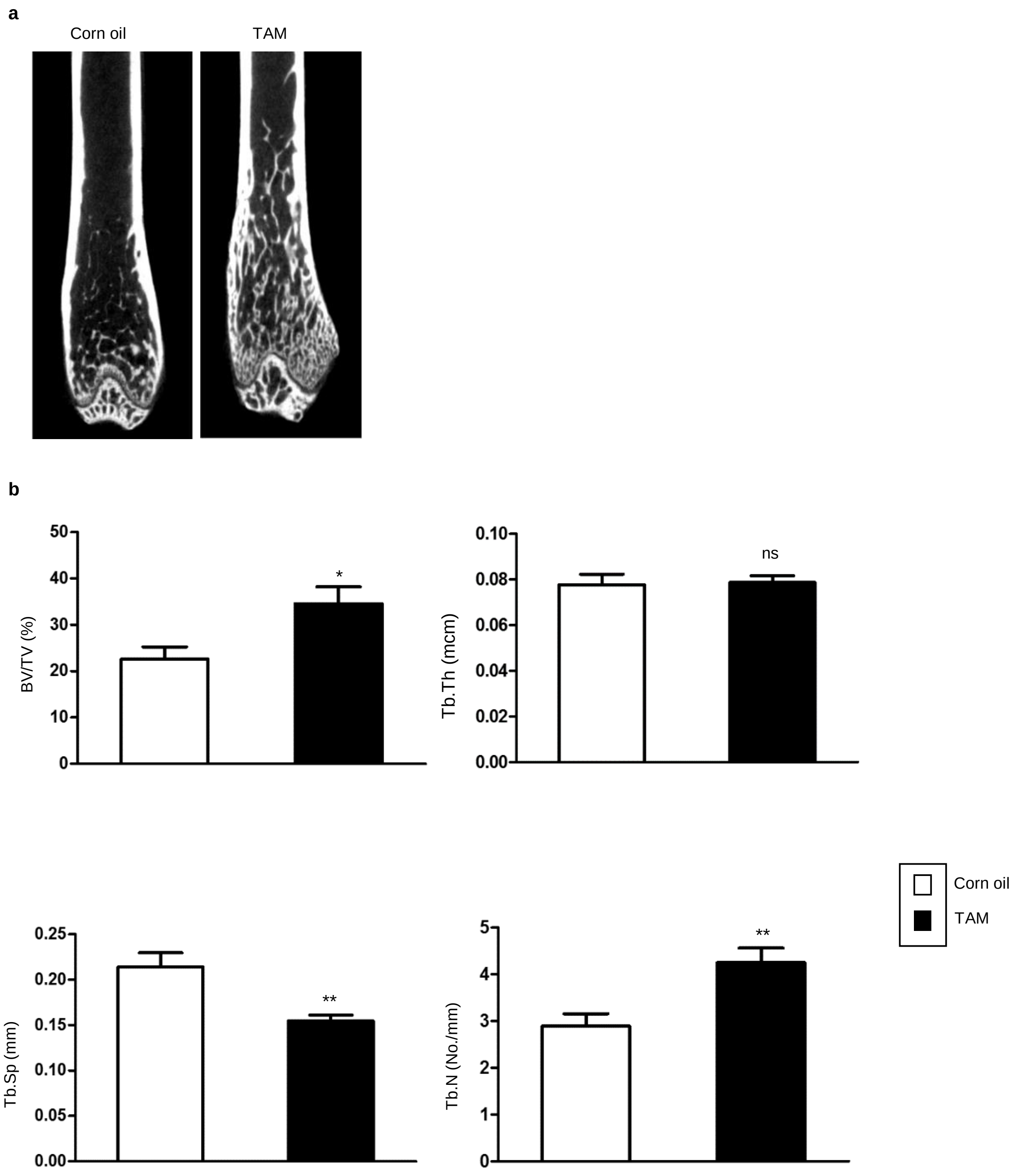

Supplementary Fig. S2: TAM administration increased bone mass in normal adult mice.
(a) Representative micro-CT images of femur bones from C57BL/6 mice showing the effect of TAM on bone mass. TAM and corn oil were injected to age- and sex-matched littermates on every other day for 3 doses. One month after the last injection, bone tissues were retrieved and analyzed. TAM-injected bones showed a significant increase in bone density compared to corn oil-injected bones. (b) Micro-CT quantification of several bone indices revealed high trabecular bone mass in TAM-injected mice. Increase in BV/TV, bone volume/tissue volume (%); Tb.N, trabecular number (No./mm); while decrease in Tb.Sp, trabecular spacing (mm) were found in TAM-injected bones. But no significant change in Tb.Th, trabecular thickness (mcm) was observed. N=6 mice per group. *P < 0.05. **P < 0.01. ns = Non-significant.

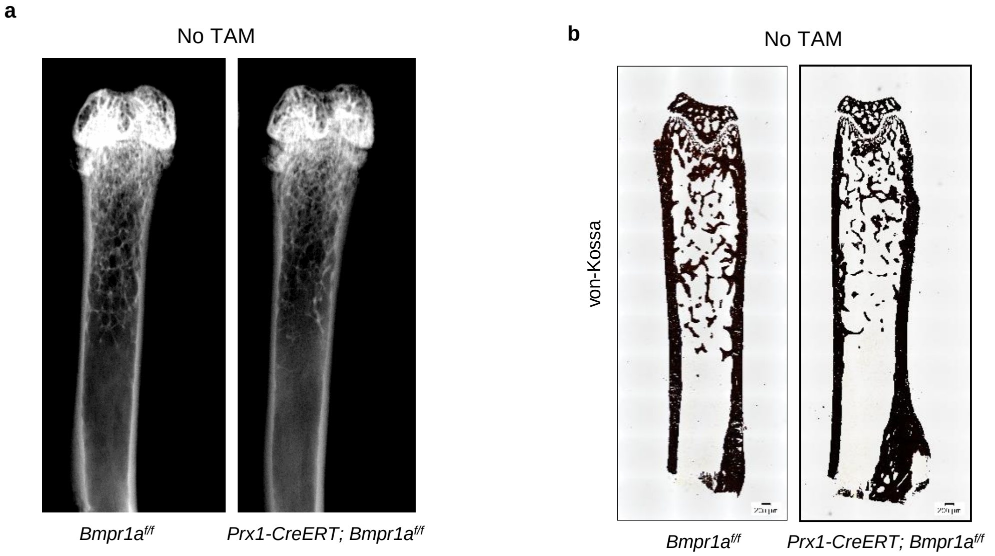

Supplementary Fig. S3: Radiograph imaging and histological examination of femurs from *Prx1-CreERT; Bmpr1a$^{f/f}$* and *Bmpr1a$^{f/f}$* mice treated with no tamoxifen.

(a) X-Ray analysis of long bones from *Prx1-CreERT; Bmpr1a$^{f/f}$* and *Bmpr1a$^{f/f}$* mice showed no difference in the radiodensity in the absence of tamoxifen injection. N=4 mice/group.

(b) Longitudinal, undecalcified sections of the distal femurs were stained with the von-Kossa method. The results revealed no difference in trabecular bone mass between *Prx1-CreERT; Bmpr1a$^{f/f}$* mice and their respective littermates treated with no tamoxifen. Scale bar = 200µm. N=4 mice/group.

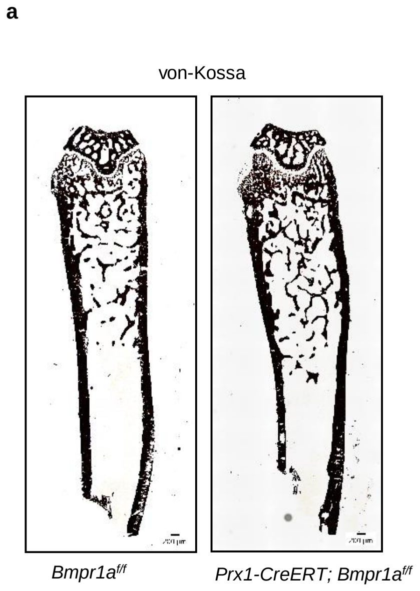

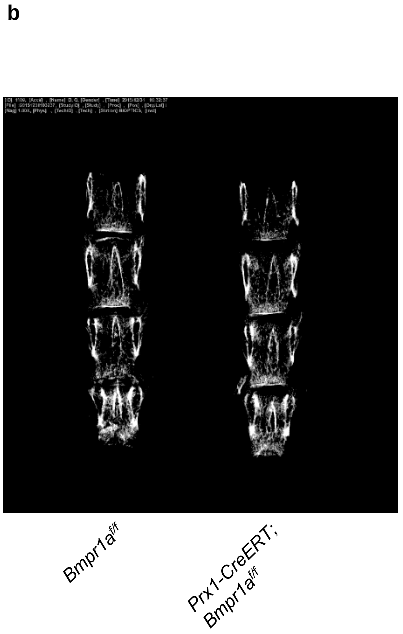

Supplementary Fig. S4: Assessment of the amount of mineralized bone and radiograph imaging for lumbar vertebrae from *Prx1-CreERT; Bmpr1a$^{f/f}$* and *Bmpr1a$^{f/f}$* mice.

(a) Longitudinal, undecalcified sections of the distal femurs were stained with the von-Kossa method. The results revealed increased amount of mineralized bone in *Prx1-CreERT; Bmpr1a$^{f/f}$* mice than their respective littermates. Scale bar = 200µm. N=8 mice/group. (b) X-Ray analysis of lumbar vertebrae showed no difference in the radiodensity in *Prx1-CreERT; Bmpr1a$^{f/f}$* and control mice. N=4 mice/group.

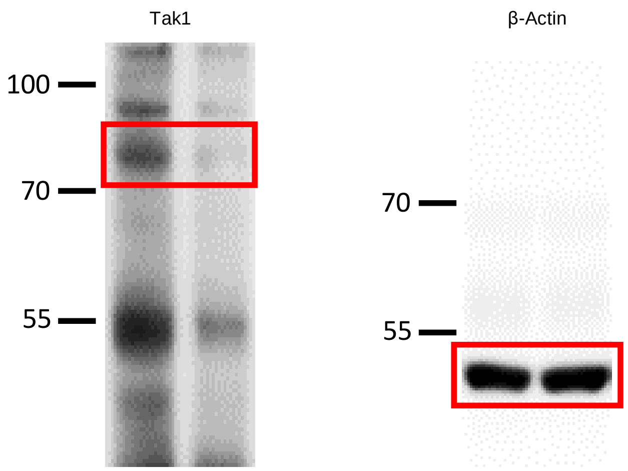

Supplementary Fig. S5: Full-length blot for Fig. 7a

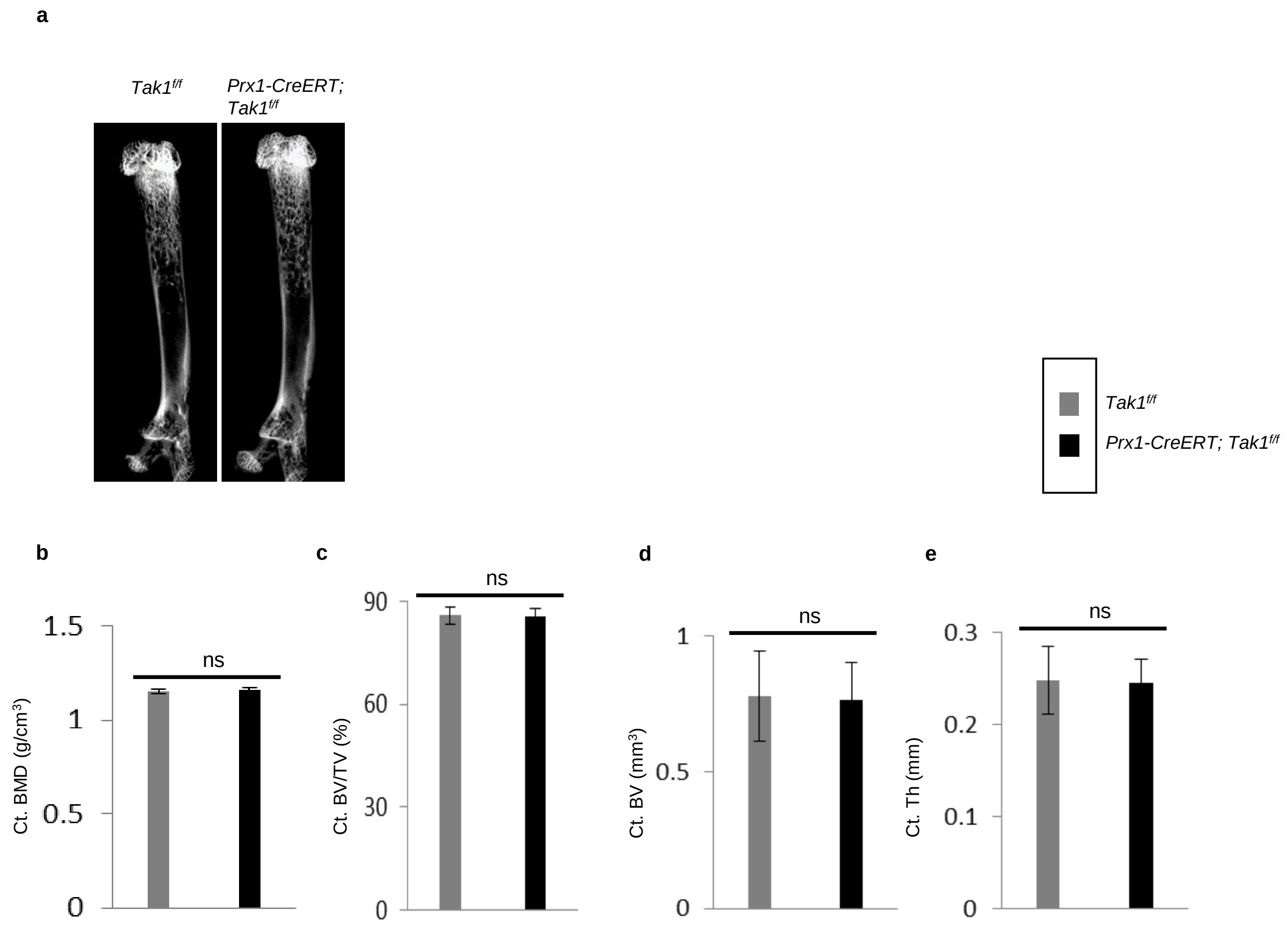

Supplementary Fig. S6: Radiograph of femurs and micro-CT derived cortical bone morphology parameters from *Prx1-CreERT; Tak1$^{f/f}$* and *Tak1$^{f/f}$* mice.

(a) X-ray analysis of long bones showed no significant change in bone mass phenotype between *Prx1-CreERT; Tak1$^{f/f}$* mice and WT littermates. N=4 mice/group. (b-e) Micro-CT derived cortical bone morphology parameters revealed no significant difference between mutant and control mice. Ct. BMD, Cortical bone mineral density (g/cm^3) (b); Ct, BV/TV, cortical bone volume/tissue volume (%) (c); Ct. BV, cortical bone volume (mm^3) (d); and Ct.Th, cortical thickness (mm) (e). N=6 mice per group. ns = Non-significant.

a

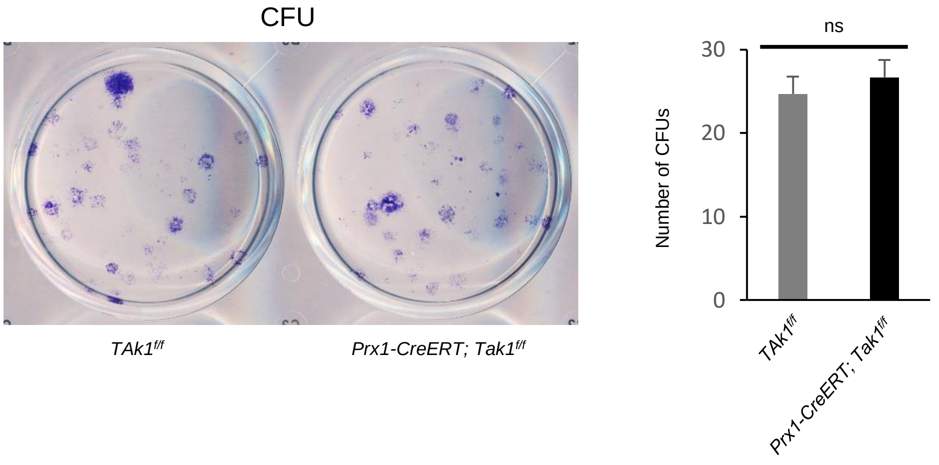

b

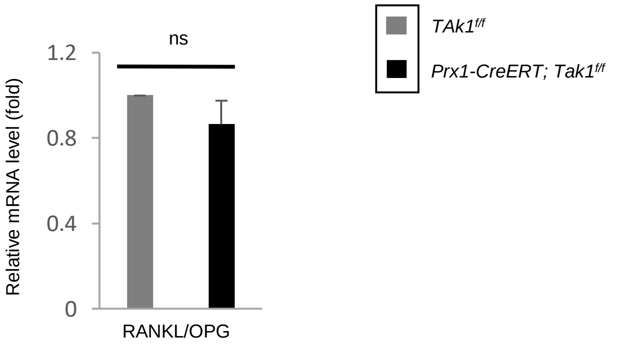

Supplementary Fig. S7: Bone marrow colony forming units (CFU assay) and mRNA level of Rankl to Opg ratio from BM-MSC of *Prx1-CreERT; Tak1$^{f/f}$* and *Tak1$^{f/f}$* mice revealed no significant difference between mutant and control mice.

(a) In vitro CFU assay carried out from harvested bone marrow MSCs of *Prx1-CreERT; Tak1$^{f/f}$* and control littermates resulted no significant difference in the number of ALP-positive CFUs. Left panel: ALP staining. Right panel: quantitation data. N=3 mice per group, ns = Non significant. (b) Quantitative RT-PCR analysis of Rankl and Opg mRNA. Results showed no significant difference in the ratio of Rankl to Opg in *Prx1-CreERT; Tak1$^{f/f}$* and control mice at adult stage. N=4 mice. ns = Non significant.

Supplementary Table1: Comparative study of our MSC-specific Bmpr1a and previously reported osteoblast/osteocyte-specific Bmpr1a knockout mouse lines

	BM	Ct.M	BR	BF	Ref.
Prx1-CreERT; Bmpr1a$^{f/f}$	⬆	⬆	⬆	⬇	
Osteocalcin-Cre; Bmpr1a$^{f/f}$	⬆		⬆	⬇	21
Col1-CreERT; Bmpr1a$^{f/f}$	⬆		⬆	⬇	29
Dmp1-Cre; Bmpr1a$^{f/f}$	⬆	⬇	NC	⬆	31

BM = Bone Mass, Ct.M = Cortical bone Mass, BR = Bone resorption, BF = Bone Formation, NC = No change, ⬆ = increased, ⬇ = decreased
